# Supplementary figures and images for: Bioindicator snake shows genomic signatures of natural and anthropogenic barriers to gene flow
Source: PLoS One. 2021 Oct 29;16(10):e0259124. doi: 10.1371/journal.pone.0259124 (PMC8555784; doi:10.1371/journal.pone.0259124)

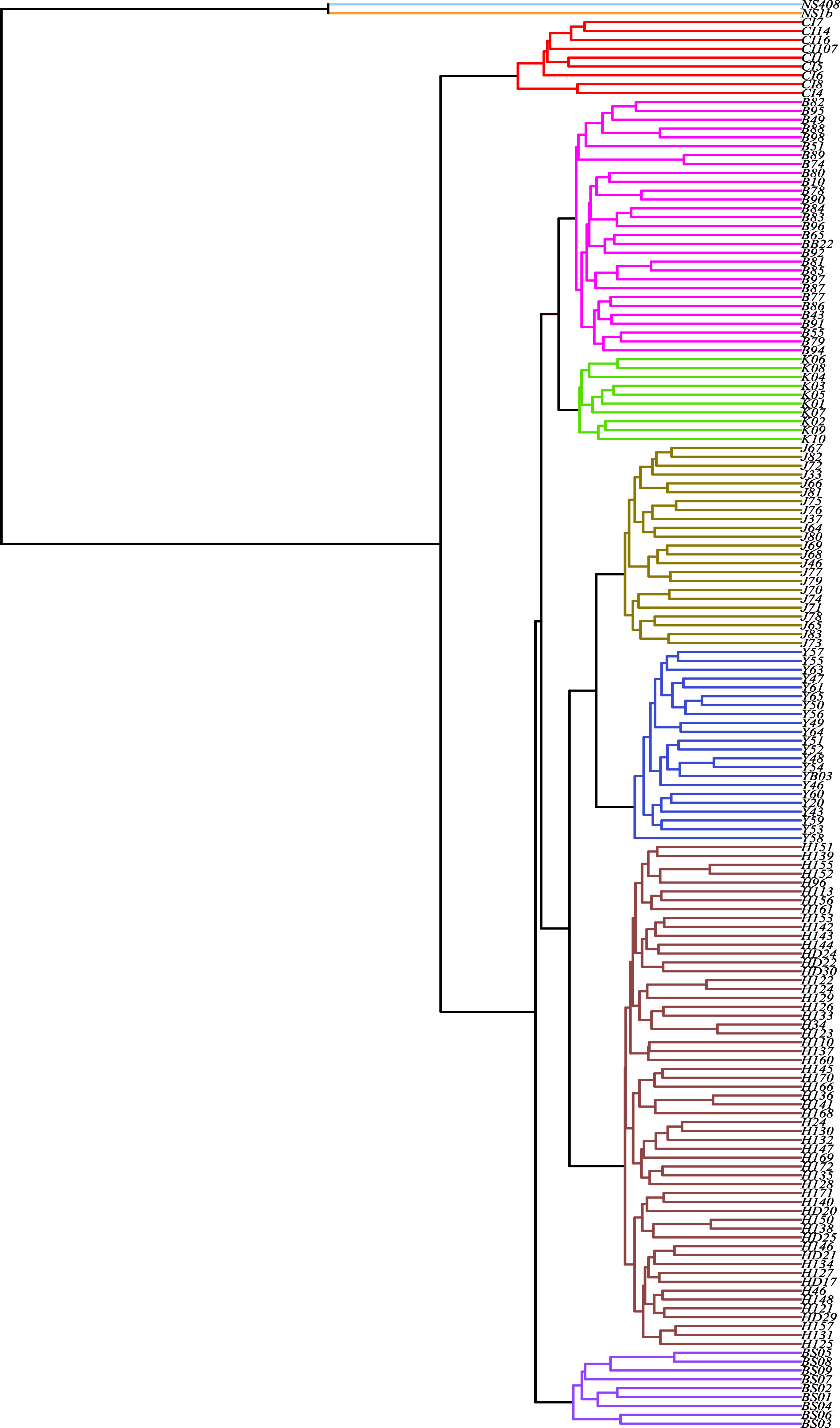

Supplement: S1 Fig — Calculated using 4688 single-nucleotide polymorphisms from across the genome. Note that these relationships do not necessarily reflect a true phylogeny. (TIF) [file pone.0259124.s001.tif]

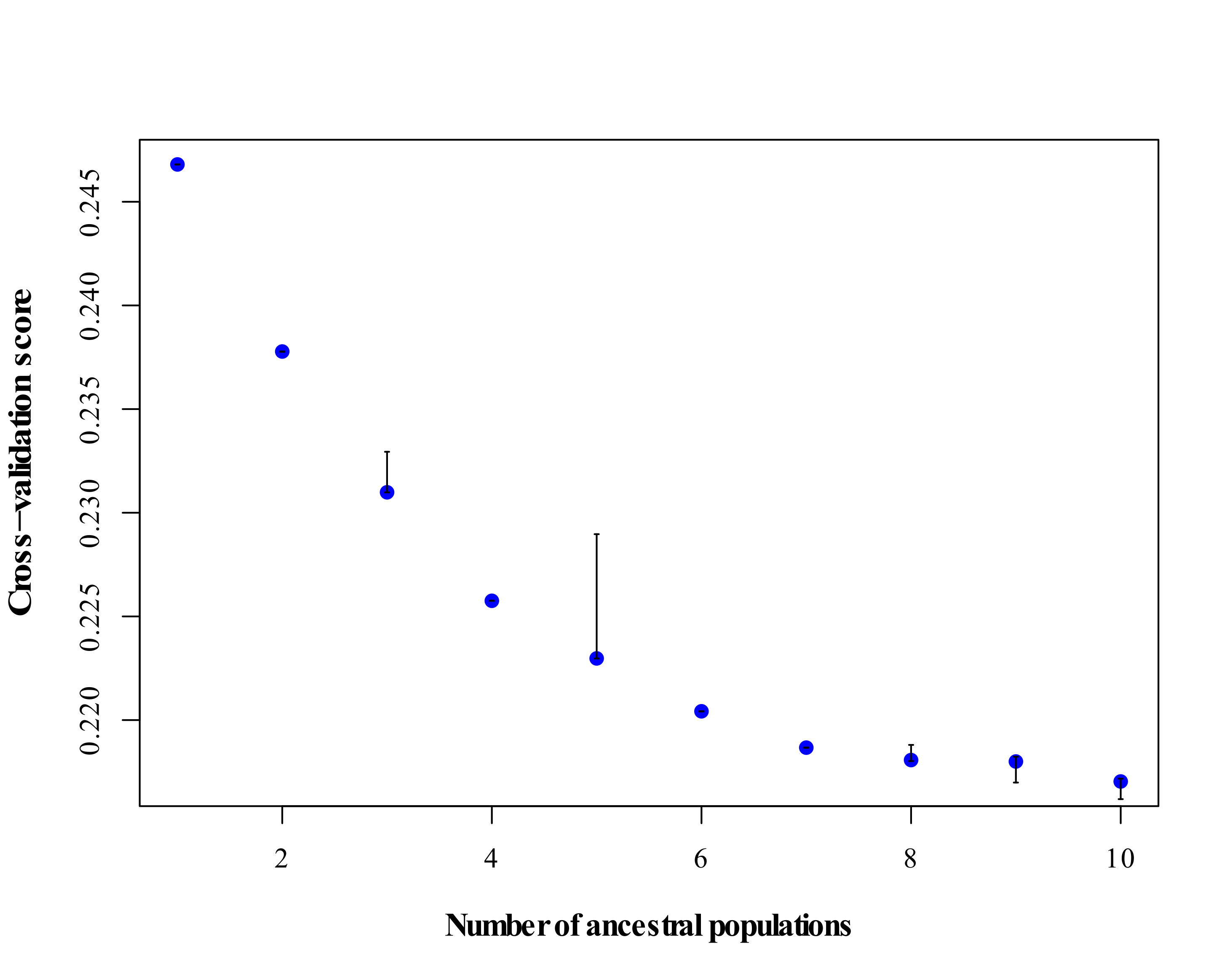

Supplement: S2 Fig — Lower values of the cross-entropy criterion indicate a better fit to the data. (TIF) [file pone.0259124.s002.tif]

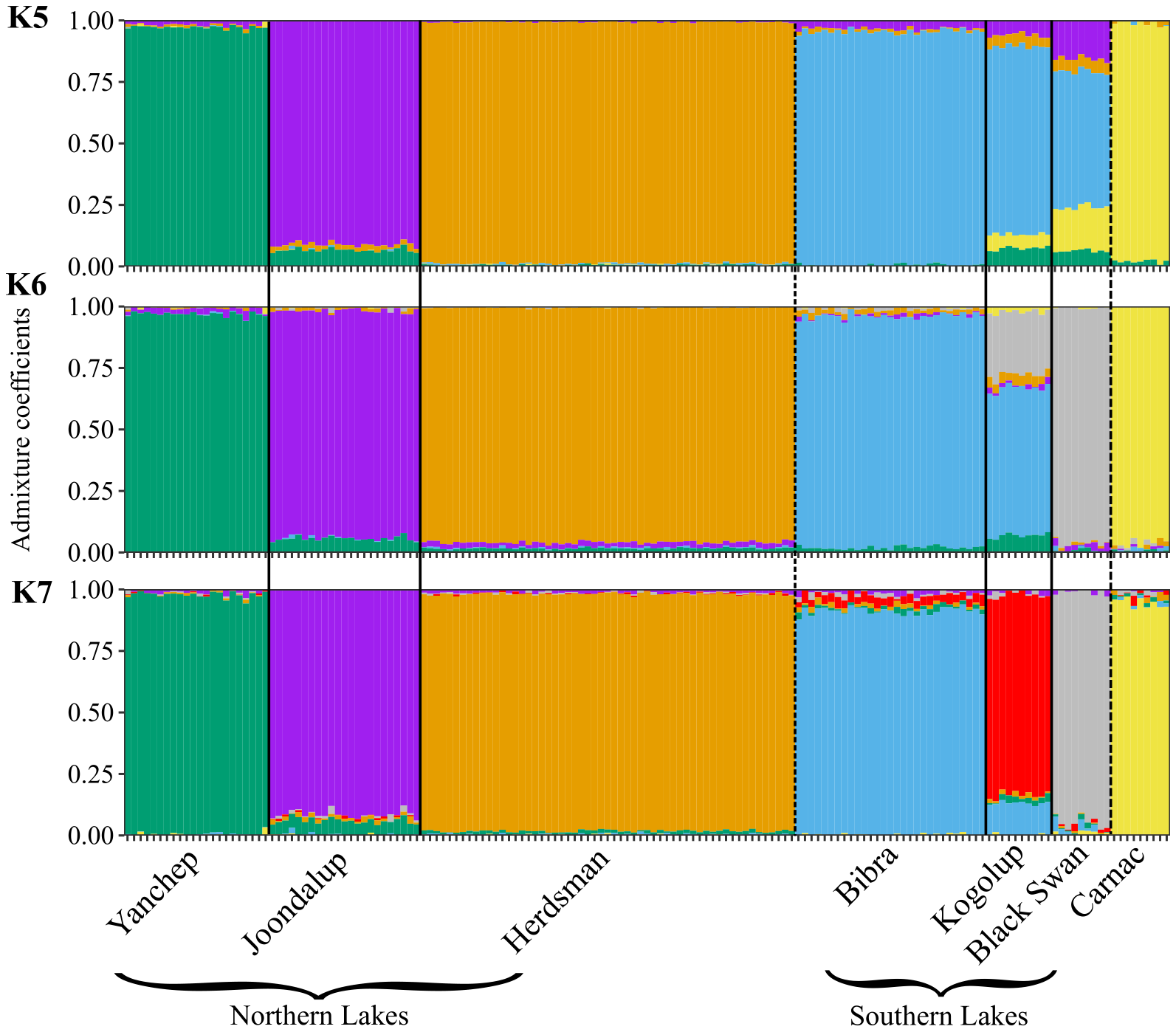

Supplement: S3 Fig — Each tick mark on the x-axis represents an individual snake, which are grouped by sampling locations. The dashed line represents the biogeographic barrier of the Swan/Canning Rivers separating the northern and southern sampling localities. The y-axis represents the fraction of individuals’ genome that originates from a particular ancestral population, each of which has been given a unique colour. (TIF) [file pone.0259124.s003.tif]

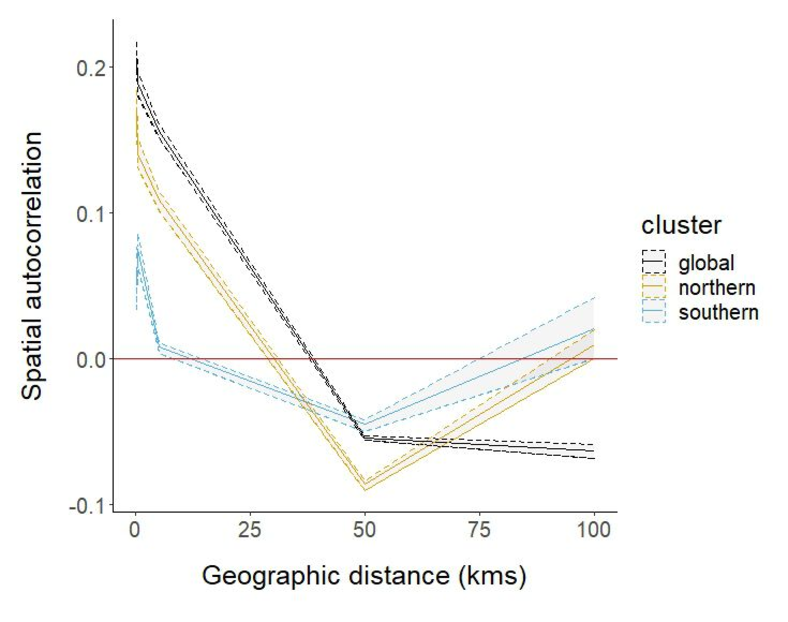

Supplement: S4 Fig — Cluster indicates the population used for analysis. Global is all mainland snakes, Northern and Southern are the populations either side of the Swan/Canning River system. The probability value at each distance class shows the proportion of permuted r values greater than the observed value in that distance class, based on 999 permutations of the SNP by sample matrix. (TIF) [file pone.0259124.s004.tif]

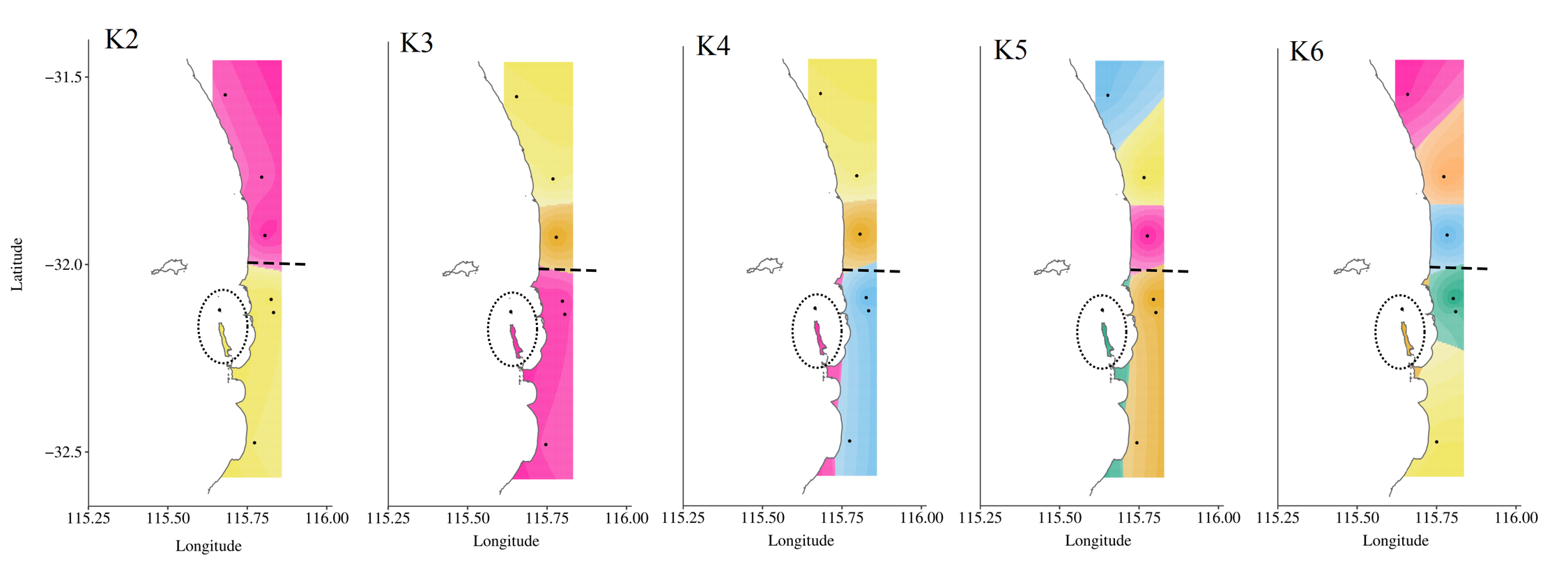

Supplement: S5 Fig — Unique colours in each panel represent an ancestral cluster. Black points indicate sampling sites. The dashed line represents the Swan/Canning River systems, while the dashed ring outlines Carnac and Garden Islands. (TIF) [file pone.0259124.s005.tif]
