# Supplementary material for: Bioindicator snake shows genomic signatures of natural and anthropogenic barriers to gene flow
Source: PLoS One. 2021 Oct 29;16(10):e0259124. doi: 10.1371/journal.pone.0259124 (PMC8555784; doi:10.1371/journal.pone.0259124)
Supplement: S1 Table — (DOCX) [file pone.0259124.s006.docx]

Table S1: Tukey HSD pairwise post-hoc test comparing body condition among six populations of *Notechis scutatus occidentalis* around Perth, Western Australia.

| Sites | Estimate | SE | Df | t-ratio | *p* |
| --- | --- | --- | --- | --- | --- |
| Bibra Lake – Black Swan Lake | -13.24 | 11.9 | 76.3 | -1.12 | 0.87 |
| Bibra Lake – Herdsman Lake | 43.49 | 15.4 | 76.6 | 2.82 | 0.06 |
| Bibra Lake – Lake Joondalup | 18.71 | 16.6 | 76.5 | 1.13 | 0.87 |
| Bibra Lake – Kogolup Lake | 0.83 | 11.3 | 76.1 | 0.07 | 1.00 |
| Bibra Lake – Yanchep | 23.60 | 20.7 | 76.9 | 1.14 | 0.86 |
| Black Swan Lake – Herdsman Lake | 56.73 | 18.1 | 76.9 | 3.14 | **0.03** |
| Black Swan Lake – Lake Joondalup | 31.95 | 19.1 | 76.8 | 1.68 | 0.55 |
| Black Swan Lake – Kogolup Lake | 14.07 | 13.8 | 76.5 | 1.02 | 0.91 |
| Black Swan Lake – Yanchep | 36.84 | 23.2 | 77.0 | 1.59 | 0.61 |
| Herdsman Lake – Lake Joondalup | -24.79 | 11.2 | 76.0 | -2.21 | 0.25 |
| Herdsman Lake – Kogolup Lake | -42.67 | 17.5 | 76.3 | -2.44 | 0.16 |
| Herdsman Lake – Yanchep | -19.89 | 11.9 | 76.8 | -1.67 | 0.55 |
| Lake Joondalup – Kogolup Lake | -17.88 | 18.4 | 76.3 | -0.97 | 0.93 |
| Lake Joondalup- Yanchep | 4.89 | 14.5 | 76.5 | 0.34 | 0.99 |
| Kogolop Lake – Yanchep | 22.77 | 22.3 | 76.7 | 1.02 | 0.91 |
