# Supplementary material for: Bioindicator snake shows genomic signatures of natural and anthropogenic barriers to gene flow
Source: PLoS One. 2021 Oct 29;16(10):e0259124. doi: 10.1371/journal.pone.0259124 (PMC8555784; doi:10.1371/journal.pone.0259124)
Supplement: Attachment — Submitted filename: PLOS One reviewer responses.docx [file pone.0259124.s007.docx]

General comments

Jeremy Larroque: **While the results provide new information on the potential connectivity of this species, the authors tend to over-interpret the results, discussing non-significant results and ignoring the small sample size in half of the populations. There are also a number of concerns regarding the mapping and SNPs detection.**

**Many aspects of the methods are not sufficiently described. All the sections describing a method need to start with the goals of the method, so we know why we are taking all of these steps. There are also a number of concerns regarding the SNPs detection with, in particular, some filtering steps missing, which makes population genetics analyses unreliable.**

**The Results section is correct, but where are the sequencing results: what is the mapping success, what are the number of SNPs removed at each step of the filtering, what was the repeatability, etc?**

**You are over-interpreting your results, the limited sample size must be discussed. Additionally, it is not clear how the results can be useful for the species management.**

We thank Jeremy for his helpful constructive criticism. However, we do not agree with some of the major comments. Primarily the ‘small sample size’ and unreliable results.

We have now substantially updated the Methods section based on all reviewer comments. We believe the filtering to be reliable and had already accounted for many of the potential issues raised by the reviewer.

Specifically, we have not included the number of SNPs removed at each step, and we don’t see the value of reporting this information but are happy to amend it if justified. We will be providing all data and R script upon publication so these data will be available.

DArTseq reproducibility scores were used in the filtering pipeline and this is specified. DArTseq processes and QC are very robust, and commonly used in modern literature. Therefore we feel we should not have to go into detail of their methods.

We believe the sample size is reasonable and adequate for the analyses we have undertaken. We have been careful not to extend our interpretations beyond the limits of the dataset. A sample size of 8-10 individuals is reasonable for interpretation. Please see more detailed comments and literature in the responses.

Reviewer 2: **This is a very interesting study that describes the effects of urbanization on tiger snake population genomics in and around Perth to inform conservation strategies. It offers a very strong argument for protection of the remaining wetland habitat, especially in the northern part of the species’ range. The authors use RAD-associated SNPs to elucidate population differentiation and effective population size as well as to look for a correlation between heterozygosity and body condition as a proxy for fitness. Overall, the article is well-written and well thought out, with a few major issues related to interpretation that need to be addressed.**

**First, the authors refer to the urban island population of Herdsman as completely isolated, but with the data they present, they can only infer that this is a highly differentiated population, not that there is no gene flow connecting it to the other populations. So, they should please be careful not to refer to “total isolation”. One interesting way that the authors could address the question of gene flow is to test different metapopulation structure and migration models with a program such as Ima2, FastSimCoal, or Migrate-N.

The second major issue is the discussion about the link between heterozygosity in these RAD-associated SNPs and body condition, which the authors use a proxy for fitness. First, I wonder why the authors did not use the inbreeding coefficient instead of heterozygosity to test for the relationship, because they are looking for evidence of inbreeding depression, and the inbreeding coefficient measures identity-by-descent, which is a measure of inbreeding. The two values (Het and FIS) are related but not the same. For example, for Herdsman Lake and Lake Joondalup, Het = 0.09, but the FIS is higher for Herdsman Lake. Also when discussing this (around Line 317 and on), the authors talk about whether or not the loci are in coding regions of the genome, and whether or not they are under selection (maybe linked to genes under selection?). This is something that they can test by doing an Fst outlier analysis, which should be done anyway because NeEstimator assumes that the loci are evolving “neutrally”, i.e. genetic drift is the only source of the signal in the data.

Another issue is with the interpretation of the pattern seen in the Carnac Island population. Interestingly, this population has a lot of private alleles. It’s not clear as written what the connection is between phenotypic plasticity and the presence of these private alleles. I made several suggestions for how to reformat this discussion point. Related to this, the authors should please show the graphs for higher K values. Based on the cross-validation plot, the least error comes at K = 7, but the authors only show up to K=4. At high K values, do you see admixture in the Carnac Island population? Even if not, it’s still possible that these snakes came from an unsampled population. Also, it’s interesting that even at K=4, the Herdsman cluster is still distinct with no evidence of admixture. What about at higher K values? This population does indeed appear very isolated as the authors point out! But still, I wouldn’t refer to it as receiving no migrants without explicitly testing for migration rates (see above).**

We thank the reviewer for their positive feedback and helpful comments. Although we have not conducted all suggested analyses – see specific comments for details – we found a very strong correlation between individual heterozygosity and inbreeding levels, and no correlation between inbreeding levels and body condition (added to the ms). We have added the results from outlier tests and higher K values.

Miss-interpretation of results have now been revised according to suggestions.

**Abstract**

**Line 35 – “Ne” should appear line 30, when you mentioned the “effective population size” for the first time.**

Added to line 30

**Line 39 – How your results can prevent demographic changes?**

We have removed the vague statement about demographic changes, and modified this sentence to say “If tiger snake populations continue to decline in urban areas, our results provide a baseline measure of genomic diversity, as well as highlighting which ‘islands’ of habitat are most in need of management and protection.”

**Introduction**

**Line 51 – I suggest giving the definition of genetic drift here instead of line 56: “levels of genetic drift (i.e., stochastic loss of alleles through time)”.**

Added to lines 51-52

**Line 61: Please change to “…led to the loss of 70% of the original…”**

Added to line 61

**Line 73: Please add “Prior to urbanization,….”, to put this sentence in context. Also, please add an estimate of the time since urbanization, i.e. nearly 200 years, or since British colonization, etc.**

Added to lines 61 & 73

**Lines 84-85 – “comparing the effective population size… …to explore the importance of population size” seems a bit redundant.**

We have changed this sentence to read “…calculating and comparing the effective population sizes across populations to explore the impacts of genetic drift and isolation, and compare individual heterozygosity to a body condition index…”

**Line 85: Please change this sentence from, “Analyses included calculating and comparing the effective population size among populations to explore the importance of genetic drift, population size and isolation; and comparing individual heterozygosity to a body condition index [33, 34] to investigate the potential influence of genetic degradation on the fitness of populations,” to this:
“Analyses included calculating and comparing effective population sizes across populations to explore the impacts of genetic drift and isolation and comparing individual heterozygosity to a body condition index to investigate the potential relationship between fitness and heterozygosity”. Because you do not calculate the census population size, the way it is currently written is confusing. Also, I would not use the term “genetic degradation”.**

We have updated the sentence to read “Analyses included calculating and comparing the effective population sizes across populations to explore the impacts of genetic drift and isolation, and compare individual heterozygosity to a body condition index (Gibbs and Chiucchi 2012; Moss et al. 2019) to investigate the potential relationship between fitness and heterozygosity.”

**Line 88 – You should add a reference to Fig 1 here.**

Added to line 87

**Methods**

**Line 96: Please delete the first sentence, as it’s redundant with the intro. Also, “landscape coverage of wetlands” is not worded clearly.**

We have removed the sentence.

**Lines 97-99 – Please add the sample size for each population, and is it possible to cite the populations from north to south, it is easier to follow on Fig 1.**

We have reordered the sites north to south and added the sample size for each.

**Line 98: fix typo (“the later” should be “the latter”)**

This word has been removed.

**Line 133 – “149 samples”, what about the two eastern snakes? Have they been sequenced in the same way?**

We have updated the sentence to say 161 samples.

**Lines 136-142 – Please explain why you filtered for these parameters?**

**You must also remove loci in high linkage disequilibrium as they add minimal extra information and could overly influence some methods (Price et al., 2008; Zou et al., 2010), loci showing deviation from expected Hardy–Weinberg equilibrium as they can be indicative of genotyping errors (Anderson, Epperson, et al., 2010; Anderson, Pettersson, et al., 2010), null alleles (Brookfield, 1996), or selection (Wittke‐Thompson, Pluzhnikov, & Cox, 2005) and loci potentially under selection as you are quantifying the neutral evolutionary processes of gene flow and genetic drift (Beaumont & Nichols, 1996). These missing steps make me wonder about the reliability of all the following analyses.**

We have already accounted for linkage disequilibrium by retaining only a single SNP from each RAD locus. This is common practice in many ddRAD datasets and adequately accounts for LD by removing SNPs in close physical proximity. We have now clarified this in the Methods section of the manuscript. With respect to filtering on HWE, we do not believe this is a useful step for RADseq data (e.g. not filtering on HWE is likely to be better than many commonly-used HWE filtering techniques, with potential for major problems with inferring population structure <https://doi.org/10.1101/2021.06.15.448615>). Sequencing or library preparation artefacts as well as potential paralogs have been accounted for in a number of other filtering steps (e.g. standard DArTseq pipeline quality thresholds, minor allele counts and read depth thresholds) and so we do not believe these to be an issue.

We are aiming to quantify overall patterns of the distribution of genomic diversity across the study area, which includes the small component of these patterns that is due to selection across the landscape. The outlier test shows that 2.8% of the loci are under putative selection, and these SNPs are thus unlikely to be substantially influencing the overall patterns of genomic structure anyway. We have now clarified this in the results. It is also not common practice to remove small numbers of SNPs under putative selection in population genomic studies that utilise thousands of loci across the genome.

This paragraph now reads as “We used a custom R script to prune unwanted SNPS and retain SNPs of interest. To remove potential bias due to sequencing or genotyping errors, we retained SNPs with total read depth > 10 and < 100, as well as those with high DArTseq reproducibility scores (no SNP < 100% reproducible in technical replicates). Reads that did not map to the reference genome in the DArTseq pipeline were also discarded. To account for bias due to linkage disequilibrium [40], we filtered out SNPs in close proximity to one another, by retaining just one SNP from each RAD locus. We also retained only SNPs that were genotyped in a high proportion of samples (callrate > 0.95), had a minor allele count ≥ 3, and observed heterozygosity < 0.6. Finally, we removed any sample that had > 20% missing data. This produced a large, high-quality SNP-by-sample matrix, with a low overall level of missing data (1%). Once filters had been applied we retained 4688 SNPs from 159 Western and two Eastern tiger snake individuals.”

**Lines 140 - 142: Move these 2 sentences to Results (retained SNPs)**

As the subsequent analyses are based on the filtered dataset, we believe including the results here is more useful for readers. This is common practice in current literature.

**Line 141 – “159” or 149 western snakes?**

We have clarified the number of snakes used throughout the ms.

**Lines 142-144 – Please indicate the name and the goal of this analysis. What genetic distance was used? Have you tried to bootstrap loci to have an idea of the support for this tree? Overall, I do not understand the goal of doing this analysis here as it can be considered as a clustering analysis as TESS3 used latter in the MS, could you explain please?**

We were not creating a true bootstrapped phylogeny here, which would require more complex analysis. This was just a minor step to ensure that the data showed sensible patterns relating to the spatial scale of sampling. We would have little faith in the dataset for subsequent analyses if there was no structure at all, e.g. it could indicate broad contamination of samples.

This section now reads as “As a preliminary measure to investigate whether our filtered dataset was appropriate for inferring relationships between individuals and populations, we created and visually inspected a hierarchical clustering dendrogram based on Nei’ genetic distance (Fig S1). All individuals fell within their sampled localities, and populations clustered in line with expected geographic distances and landscape barriers, including the two individuals from eastern Australia, suggesting that the retained SNP dataset was adequate for further analysis.”

**Line 148: Move the kinship results to Results**

We need to report the results of the filtering process here, as subsequent analyses are based on this dataset. We have improved the text for clarity.

“To ensure relatedness between individuals was not high enough to impact our data analysis or interpretation, we calculated pairwise kinships via the ‘beta.dosage’ function of the *hierfstat* package [40, 41]. Four pairwise values from a total of 2604 comparisons had kinships > 0.25 but < 0.3 (commonly values of 0.25 indicate full siblings), therefore no individuals were removed.”

**Line 153: What version of adegenet did you use?**

We removed mention of this package as it was not relevant to our reported methods.

**Lines 154-156 – While I agree it is important to provide the name of the functions and packages used for reproducibility of analyses, you must first provide the name and the purpose of the analysis. What does the “cmdscale” function? Why did you choose the Prevosti distance? What is a “multidimensional scaling plot”? Why doing individuals and populations analyses?**

**Please explain, not all the readers of Plos One are experts in genetics. Same comment for the G”st.**

We have substantially updated the wording of this paragraph, including removing the “and populations” that was causing confusion.

There are a variety of distance measures and the number of allelic differences between individuals is a commonly used metric. Prevosti's distance is a well-known example of this, which dates back to the 1970s (Prevosti et al., 1975). While many manuscripts do not specify the type of genetic distance measure used, we have chosen to be explicit in presenting this information. We could similarly have used Dice, Jaccard, Manhattan, or GenAlEx genetic distances.

We have stated that GST is an FST analogue that corrects for the number of subpopulations being considered, and that it is a measure of population differentiation. This should be enough for readers to understand that it is a metric of the level of difference between two groups of samples.

We have modified the methods throughout the ms to improve clarity.

**Line 158: What version of mmod?**

Added to line 164

**Line 161/166: Please be consistent with how you italicize K, (italicized in 161 and not in 165)**

Fixed throughout the ms

**Line 163: What version of tess3r?**

Added to line 169

**Lines 160-167 – Same comments, I would like something like that “With the aim to explore the genetic structure across populations and to assess potential gene flow among populations, we searched for genetic groups using TESS3, a spatially explicit ancestry estimation method implemented in the tess3r package in R. The number of ancestral populations (K) was evaluated using XX independent runs of each K with ZZ < K < YY. A cross‐validation procedure was used to select the best value of K according to the asymptote in the plot of cross-validation scores. etc”**

We have used some of these suggestions and rejigged our methods throughout the ms. We have not followed the exact formatting, however, as we prefer our style of writing.

**Line 165: With cross-validation scores, I think you look for the plateau or minimum value rather than the biggest drop – could you please also plot K=5, 6, and 7? The plot doesn’t plateau until at least 7. Also, please move the discussion of the best K values to the Results section.**

We have now created these additional plots and presented the additional K value results in the supplementary material. At these higher levels of division, the high level of power available in such large SNP datasets simply splits individuals into their sampling localities, which undermines the point of the analysis (to infer ancestral relationships between populations). We feel a figure of all K values would be too confusing for readers to interpret.

As subsequent analyses are based on selecting set K values, we feel it is important to include report the best levels of K in the methods.

The methods now read “To explore genomic structure and assess potential gene flow among populations, we searched for genomic groups using the TESS3 algorithm – a spatially explicit ancestry estimation model from the *tess3r* package (version 1.1) [46, 47]. The ‘tess3’ function incorporates the latitudes and longitudes of each sampled individual to account for the influence of isolation-by-distance on ancestry coefficients, with large drops or plateaus in the scree plot identifying appropriate values of *K* (Fig S2). We considered ancestry coefficients for *K* values of 2, 3 and 4 as these values showed the largest improvements in cross-entropy, with *K* = 4 showing a substantial change in slope, and plotted these for each individual. For exploratory purposes we also plotted ancestry coefficients of *K* = 5, 6 and 7 (Fig S3). To confirm whether there SNPs under putative selection were driving patterns of population structure, we conducted a genome scan for outlier loci using the ‘pvalue’ function. This outlier test uses overall differentiation to discern if a portion of SNPs have greater allele frequency differences than expect from a neutral distribution [48]. We used *K =* 4 as we considered this the most useful for inference, and a Benjamini-Hochberg correction to achieve a false discovery rate of one in 10 000.”

**Lines 165-167 – This belongs to the Results section.**

See comment above.

**Line 170: Which GenAlEx version?**

Added to line 185.

**Line 171 – What is Ae? Is it the rarefied allelic richness of El Mousadik & Petit (1996)?**

We explain the abbreviation at first use as the “effective number of alleles”. Allelic richness is typically presented as Ar.

**Line 192: Which version of lme4?**

Added to line 207

**Line 176: Please replace “9” with “nine”**

Added to line 191

**Lines 178-190 – I do not know anything about snakes’ body condition, my only concern would have been about seasonality, but you removed the Carnac population, so everything looks fine to me.**

Thank you.

**Line 183: Please define “SVL” for people who aren’t familiar**

Added to line 198

**Line 190: Lo and bSMA should be in Results**

Again, we use those numbers in the equation presented in the methods, so the values are also best presented in the methods.

**Line 193 – Why include the sex as a random factor and not a fixed one?**

As we were not interested in the sex-related difference of body condition, so we wanted to account for them. The sentence now reads “with SMI as the response variable, individual heterozygosity and site as fixed predictor variables and to account for sex-biased differences, sex as a random factor.”

**Lines 201-203 – I am not sure to understand what you mean with this ratio? It is Ne/Nc=0.1? You do not seem to give any results about it in the Results section, so I suggest to remove it. If you want to estimate the census population size with your genetic data, you might want to look at the Close-kin mark-recapture method (Bravington et al. 2016).**

We have removed this.

**Results**

**Line 211: instead of “allelic isolation”, refer to “differentiation”**

Added to line 227

**Lines 209-215 – Have you tested the significance of the G”st values?**

We have tested the significance and all pairwise populations are significant. We added this result to the Table 1 figure “All non-zero values are significant (*p* < 0.001)”.

**Line 215: Please perform a Mantel test to explicitly test for isolation-by-distance by testing for correlations between matrices of pairwise geographic distance and genetic distance– you could use the adegenet package for that for example, or GenAlEx.**

**It could be interesting to do a global Mantel test as well as one just with the northern cluster and one just with the southern – you could then compare the slopes of the lines to quantify the difference between IBD in the northern cluster and southern.**

**You could also perform an AMOVA analysis to get an idea of the statistical significance of the Gst values and quantify the within/between population variance.**

We have performed a mantel test for all mainland snakes, and both the northern and southern cluster. The Mantel test found expected correlations between genomic differentiation and geographic distance, for mainland tiger snakes (*r*^2^ = 0.61, *p* < 0.01). Correlations were near identical between the northern cluster (*r*^2^ = 0.78, *p* < 0.01) and the southern cluster of snakes (*r*^2^ = 0.80, *p* < 0.01).

However, we don’t think the Mantel test is the most appropriate test to investigate IBD; for example, see the discussion presented in this paper:

<https://doi.org/10.1111/mec.13243>

Instead we conducted and present a spatial-autocorrelation test conducted in GenAlEx.

The methods now read as “To investigate isolation-by-distance, we calculated the multilocus spatial autocorrelation for the mainland, and subset of northern and southern populations of snakes, respectively. The spatial autocorrelation analysis was conducted in GENALEX (version 6.5) add-on in Excel [49, 50].”

The results now read as “Analysis of spatial autocorrelation identified significant values of r – the autocorrelation coefficient – persisting for distances up to 38 km (fig SX). We found a difference in the decay of spatial autocorrelation between the northern and southern clusters, with the northern cluster intercepting r = 0 at about 30 km and the southern cluster intercepting r = 0 at about 12 km.”

Added to discussion “Interestingly, the southern cluster exhibited a lack of spatial autocorrelation at 12km, compared to 30km in the northern cluster. As the geographic distances between populations in north differ from those in the south, interpretation of these differences is difficult without sampling a more even spread of distances between populations.”

**Lines 219-223 – Please give the percentage of the genotypic variation explained by the first two axis.**

We have added these results to the figure legend and line 246.

**Line 228: Please be consistent with how you format “TESS3R” (vs. tess3r on line 163)**

Have fixed throughout the ms.

**Lines 228-299 – I would not say “strong hierarchical” and “good support” but rather “a likely number of clusters 2 ≤ K ≤ 4” as there is no plateau for these values of K.**

Have fixed line 251-252.

**Line 229: Again, I think you should also show plots for higher K values, at least up to 7.**

We have plotted these in the supplementary material and briefly reported the results, please see above comments for discussing our thoughts on this. Retaining k=7 simply splits the data into the total number of sampled a-priori populations, due to the spatial distribution of available habitat and sampling patterns. The aim of this analysis is simply to show which populations are likely coming from the same ancestral lineages.

The new results read as “With finer-scale population splitting, Lake Joondalup separates from the northern lakes (*K* = 5) while Kogolup Lake and Black Swan Lake share ancestry with Carnac Island (Fig SX). At *K* = 6, Black Swan Lake separates from the southern lakes, and Kogolup Lake is only recognised as a unique different population at *K* = 7.”

**Lines 241-248 – Except for the private alleles number, the differences are very low, you are talking about a max difference of 0.06 for the He for example. Is there any way to test the significance of these differences?**

**It is usually considered that under n=25-30, population genetics estimates are not reliable (Hale et al. 2012; Flesch et al. 2018).**

A difference of 0.06 when using large SNP dataset is not unusually small. These values have been averaged across thousands of genetic markers including those with low minor allele frequencies. In reality, the values of heterozygosity and inbreeding coefficients that we find using out dataset are much more likely to correlate with ‘true’ values of heterozygosity across the genome (<https://doi.org/10.1111/2041-210X.13659>), than, for example, when using a small number of microsatellites or a SNP dataset filtered to MAF > 0.05.

Relative to the southern cluster of snakes, the difference is 25-33%, which is substantial. We have highlighted these in the results and now reads as “Observed heterozygosity, specifically, was 25 – 33% lower in the northern populations”.

Based on the mean and SEs, the sites that would differ significantly are clear. Eg. Ho from the northern sites would be significantly different compared to southern sites, but not each other. We do not think testing the significance would change the patterns or interpretation.

Please see other comments on sample size.

**Lines 257-259 – You must also say that it is the only significant difference.**

Added to line 284.

**Line 266: In the other sections you italicize Ne, and here you do not, please consistently italicize**

Fixed throughout

**Lines 266-269 – You got “INF” for the three populations with n≤10, there is clearly a sample size problem, you cannot discuss the effective population size values for these populations. Instead, I suggest to pool the populations as suggested by the PCoA and TESS results: Herdsman and Carnac are independent while Bibra, Kogolup and black Swan seem to belong to the same genetic cluster, as do Yanchep and Joonalup.**

**If the a priori populations are part of the same genetic population, that makes sense to estimate the population size of the genetic populations and that could help to solve your sample size issue. You must also talk about the large range of the estimations for the other populations.**

The infinite values were primarily occurring in the Jackknife confidence interval calculation, not the estimate of Ne. We have decided to removed the JackKnife estimation of confidence intervals as this method of calculating the CIs is not feasible with our data set and sampling design. We agree that presenting both methods appears confusing, and presenting one method is more appropriate.. The authors of NeEstimator state that “Precision of estimates [using the jackknife method] based on large numbers of loci might be substantially lower than suggested by traditional methods for computing Cis” (<https://doi.org/10.1111/1755-0998.12157>), and so we have retained the standard chi-squared parametric confidence interval calculation which works best.

Of the three populations with low sample sizes only one (Kogolup) had a mean Ne of INF. We feel the paragraph lines 388-398 has thoroughly discussed the results and small sample size with references. It is reasonable to present the Ne values and confidence intervals for the other six populations for which the estimator was able to converge on a value. Ne assumes individuals are randomly breeding, therefore pooling populations over a large area as suggested by the reviewer is a bigger violation of assumptions than potentially using slightly small sample sizes.

**Discussion**

**Lines 276 – 293: This paragraph isn’t organized clearly – I think it would be more clear if you start out talking about the N/S split, then say that the quenda followed the same pattern, likely caused by the rivers; it’s not clear if the quenda had other similarities from this paragraph as it is written. Then talk about the next level of differentiation, at K=3, Herdsman Lake is recovered as a distinct cluster, potentially due to the high level of urbanization. And, at K=4, Carnac Island is distinct. Then discuss the idea of habitat patches in the urban matrix acting like islands. Please edit the last sentence because you can’t tell definitively if the populations are in fact “closed”.**

The paragraph has now been reorganised and split into two. Lines 306 - 323.

We have changed ‘closed’ to ‘fragmented’.

**Line 284: Change for clarity, “…showed a similar pattern of genetic differentiation on either side of the major rivers”**

Added to line 311

**Lines 294-316 – Is it hard to distinguish between the different landscape class in the study area map, and thus it is hard to follow your explanations. You should provide the same map with the same spatial extent (as a supplement for example), but with only two classes: favourable vs. unfavourable habitats, that could help to understand the global connectivity pattern.**

Unfortunately we cannot assess the entire landscape as ‘favourable’, because we are still trying to determine what habitat is favourable by tiger snakes (future research). The idea of the map is to highlight urban areas vs. ‘green’ areas that tigers may persist in. So we have reduced to map colours to merge all green landscapes.

**Line 306: You have measured differentiation here as a proxy for gene flow, but you could also use a program like either IMa2 (DOI: 10.1093/molbev/msp296), fastsimcoal2 (https://journals.plos.org/plosgenetics/article?id=10.1371/journal.pgen.1003905), or Migrate-N (https://currentprotocols.onlinelibrary.wiley.com/doi/pdf/10.1002/cpbi.87) to test demographic models including historical rates of migration.**

There are a number of assumptions and priors needed to calculate migration rates. We do not have most of this information, so it would be very speculative. We don’t think assessing migration will strengthen our results.

**Lines 309-316 – This part is not clear. Is the Black Swan population part of the Beeliar park?**

In the final sentence of the paragraph we say ‘…the close relationship shared by the Beeliar wetland populations and the Black Swan population’. Which we think is pretty clear these are not the same.

**Lines 318-334 – You cannot properly estimate genetic diversity with n<25-30 (Hale et al. 2012; Flesch et al. 2018). You must talk about the very limited sample size. For example, you cannot seriously discuss almost null Fis values for which we have no idea about the significance.**

We do not agree that genetic diversity parameters cannot be calculated with n<25-30. Firstly, microsatellite studies are not very comparable to large SNP dataset studies. It is common in the literature for SNP based studies to use 4-6 individuals for differentiation, and 10 individuals for general interpretation. Please see these papers: <https://doi.org/10.1007/s10329-010-0232-4> <https://doi.org/10.1371/journal.pone.0042649>

We acknowledge that our sample sizes are small for Ne estimations, but this has been acknowledged in the in manuscript with relevant text in the Discussion section. By using thousands of SNPs across the entire genome we are able to get very accurate estimates of Fis (and other diversity estimates), which have very small confidence intervals. Many of these values have confidence intervals that do not overlap with zero. When using large numbers of SNPs, including those with small minor allele frequencies, it is expected that some of the metrics will have low values. For example, an FIS value of 0.05 on Carnac is not ‘almost null’, and likely reflects genome-wide patterns and substantial differences from populations with negative values of FIS. In reality, the values of heterozygosity and inbreeding coefficients that we find using out dataset are much more likely to correlate with ‘true’ values of heterozygosity across the genome (<https://doi.org/10.1111/2041-210X.13659>), than for example, a microsatellite dataset or a SNP dataset filtered to MAF > 0.05. Further, we have been very clear with our interpretation of low FIS values, and only highlight a potential pattern. We have now added a sentence to the Results section when discussing the FIS values, stating:

“While these values are close to zero, they likely reflect genome-wide patterns and differences between populations, with low values not unexpected when using a low minor allele frequency threshold.”

**Line 323: From this dataset, you can’t infer that Herdsman Lake and Carnac Island are necessarily completely lacking gene flow - they had the highest differentiation and FIS, but you didn’t explicitly test rates of migration, so please reword this to say “…genomic diversity was not lowest in the two sites with the highest genomic differentiation” or “highest Gst values”.**

Carnac Island snakes are completely isolated, and with our large sample size from Herdsman Lake, we believe we would have detected individuals from other populations if it wasn’t completely closed. We have more carefully worded the sentences to say “At *K* = 3, Herdsman Lake is recovered as a distinct cluster, potentially due to isolation from a sea of urban infrastructure; at *K* = 4, Carnac Island is distinct, likely due to its isolation by 7 km of ocean.”

**Lines 331-332 – What is the generation time of tiger snakes?**

We have added this into the discussion at line 363 “…such as tiger snakes, estimated at 10 years”

**Line 335: Since you calculated FIS, the inbreeding coefficient, maybe it makes more sense to test for a correlation between body condition and FIS than to use heterozygosity.**

**Or, provide a citation for using heterozygosity instead of FIS to look for a relationship with inbreeding depression (/fitness) when using RADseq SNP data.**

Thank you for the suggestion. We have added two additional tests to address this pattern. Calculating the g^2^ to confirm inbreeding signal, calculating inbreeding level (*f*) – body condition correlations, and inbreeding level – heterozygosity correlations; using the inbreedR package. The results confirm inbreeding, found a strong correlation between inbreeding level and heterozygocity, and no correlation between inbreeding level and body condition. These analyses confirm and strengthen our original results.

Added methods: “In addition, we compared body condition with *g*^2^ – a proxy for identity disequilibrium (inbreeding) [53, 59]. A *g*^2^ = 0 means no variance of inbreeding in the sample. With the *inbreedR* package (version 0.3.2) [60], we used the ‘r2_wf’ function to calculate the expected correlation between inbreeding level (*f*) and body condition, and the ‘r2_hf’ to calculate the correlation between inbreeding level (*f*) and individual heterozygosity.”

Added results: “Of the snakes with body condition data, the inbreeding among loci was significantly different from zero (*g*^2^ = 0.025 ± 0.003 S. E., *p* = 0.01). We found a high correlation between inbreeding level (*f*) and heterozygosity (*r*^2^ = 0.94), strongly suggesting that heterozygosity is a good proxy for inbreeding. Furthermore, we found no correlation between inbreeding level (*f*) and body condition (*r*^2^ = 0.07).”

Added discussion: “We found a strong correlation between inbreeding (*f*) and heterozygosity, justifying our use of heterozygosity as a proxy for inbreeding.”

**Lines 338-340 – I agree, that is why you must filter for loci potentially under selection. But the most probable explanation is that there is not enough variability**

We cannot accurately determine loci under selection with 1 desert island site & 6 wetland sites differing in urbanisation pressure. We would need more replicate sites of near identical condition to accurately determine if loci are being selected for in multiple populations. However, we have conducted an outlier test. See other comments.

**Line 338: I would assume that most of the loci are neutrally evolving, but you should probably test for that because NeEstimator assumes that only genetic drift, not selection, is responsible for the signal in the data.**

**I believe that you can do a locus specific Fst test with TESS3R to look for Fst outlier loci.**

**Also please make this statement a bit more clear – in general, would you expect loci that are under selection to show lower heterozygosity than neutrally evolving loci? Why? If a heterozygous genotype gave the c competitive advantage, you could see the opposite pattern – of higher than expected heterozygosity at that locus. I would use FIS to test for a relationship with inbreeding depression instead of using heterozygosity, as mentioned above.**

We have conducted an outlier test and added text to the methods and results. We have also conducted correlations between Fis and heterozygosity, and Fis and fitness in relation to other reviewer comments. Please see these comments.

We have added to the methods: “To confirm whether there SNPs under putative selection were driving patterns of population structure, we conducted a genome scan for outlier loci using the ‘pvalue’ function [of the tess3r package]. This outlier test uses overall differentiation to discern if a portion of SNPs have greater allele frequency differences than expect from a neutral distribution [48]. We used *K =* 4 as we considered this the most useful for inference, and a Benjamini-Hochberg correction to achieve a false discovery rate of one in 10 000.**”**

We have added to the results: “The outlier test identified 131 (2.8% of 4688) SNPs as being under putative selection. These loci demonstrate significantly higher or lower among-population genetic differentiation than expected under neutrality, many of which are possibly driven by the differentiation between Carnac Island and the mainland populations. While this small number of SNPs is unlikely to have substantially influenced our observed population structure, these loci could be responsible for influencing fitness in different environments, and thus provide a basis for further study.**”**

While the outlier test is a useful addition to the manuscript, it is also likely that many traits that contribute to local adaptation are polygenic (Lind et al., 2018; Savolainen, Lascoux, & Merilä, 2013), and FST values tend to decline as the number of loci involved increases, because there are many allelic combinations that can produce optimal trait values (Josephs, Stinchcombe, & Wright, 2017). This means that the small-effect loci involved in polygenic adaptation often exhibit only subtle allele shifts, and are less likely to be found through standard FST outlier sweeps (Berg & Coop, 2014; Kemper, Saxton, Bolormaa, Hayes, & Goddard, 2014). To conduct a more complete analysis of adaptation across the genome is well beyond the scope of this study, but is a recommendation for future work, possibly using a greater number of populations to provide replication across environmental or urbanisation gradients. We believe the discussion about SNPs under selection and genotype-environment association analyses is important to consider and suggest future directions. So we have reworded and restructured this paragraph into the following two:

“Phenotypic signatures of inbreeding depression can be measured in wild populations using heterozygosity-fitness correlations. We found a strong correlation between inbreeding (f) and heterozygosity, justifying our use of heterozygosity as a proxy for inbreeding. Our model found no effect of individual genomic heterozygosity on snake body condition, despite the broad pattern of the northern cluster sharing both lower heterozygosity and lower body condition (Fig 4). Body condition is a single measurement of fitness and low heterozygosity can translate to many other measures of fitness such as reduced body size in neonates [34], higher parasitism [78] and survival probability [19]. Populations with low heterozygosity may be experiencing changes in other life history traits that could directly or indirectly affect fitness. Similar to Sovic et al. [51], our results show that body condition was strongly influenced by site. This suggests site-specific environmental stressors such as differences in prey availability [79], anthropogenic disturbance [80] or physiological changes from bioaccumulation of contaminants [32, 81, 82] are probably more important factors than heterozygosity for reducing body condition in tiger snakes from our study sites.

In addition, our use of genome-wide loci, possibly includes many loci in non-coding regions of the genome [83], which would possibly conceal the signal from SNPs under selection. The outlier test identified 131 loci that are potentially influencing fitness in different environments in the Perth region, and these candidate loci can be investigated in future analyses. However, as many traits that contribute to local adaptation are polygenic and may not exhibit high FST values, we suggest that a genotype-environment association analysis would be a better method for investigating adaptive genomic architecture [87, 88]. Further investigation sampling tiger snake populations across their entire distribution – covering a range of native and urban wetland areas – would help identify SNPs that play a role in urban adaptation.”

**Lines 358-360 – You are overinterpreting the results, the only significant body condition difference is between Herdsman and Black Swan, with Herdsman having the lowest body condition while it is not the most northern population. The body condition seems to be linked to the local environment and not related to the location in the species range.**

We don’t believe we are over-interpreting the results, and have been very clear with the language used. The broad pattern of Northern vs. Southern heterozygosity and body condition is not likely to be coincidence and the pattern is worth of discussion. We acknowledge the lowest body condition from Herdsman lake snakes is most likely linked to environmental conditions. However, the overall level of adaptive capacity of the northern population is lower and these populations are under more urbanisation pressure. So we believe it is worth highlighting the low heterozygosity for future urban landscape management. The speculation is in the discussion and the wording around the significant values is clear in the results.

We have amended the final sentence to read “This suggests site-specific environmental stressors such as differences in prey availability [84], anthropogenic disturbance [85] or physiological changes from bioaccumulation of contaminants [32, 86, 87] are potentially probably more important factors than is heterozygosity for influencing reducing body condition in tiger snakes from our study sites.”

**Line 358: Please do not refer to “lowest adaptive potential” because with these data, you don’t know that – it’s not clear what the relationship between adaptive potential and heterozygosity at these RAD SNPs is.**

**They already have the lowest heterozygosity and poorest body condition, that’s enough for us to be worried about them, especially with increasing urbanization and isolation.**

It is universally recognised that the evolutionary potential of a population is proportional to its amount of genetic diversity. We have mentioned this in the introduction and believe it’s the most suitable term and broadest interpretation of our results. We have amended the sentence to read “The northern SCP population already shows the lowest genomic diversity – hence adaptive potential – and poorest body condition…”

**Lines 362-395 – This is an overinterpretation of non-reliable results. “An infinite estimate means either that there is no evidence for drift in that population, the sample size used is too small, or the Ne is actually large (>1000) and we are unable to differentiate which explanation is true.”, with n=9, the most probable explanation is that the sample size is too low.**

We believe the speculation around the infinite estimate for Kogolup is worthy of discussion. However, we have made the low sample size, and speculation points, more prominent. The discussion now reads as:

“The Ne estimate for Kogolup Lake population was infinite, and the lower parametric confidence interval was 3581 (Table 3). As our sample size for that population was n = 10, the infinite estimate is likely due to a small sample size; however, an infinite estimate can also mean there is no evidence for drift in that population, or the Ne is actually large (>1000). Consequently, our dataset is unable to distinguish whether or not the population is ‘very large’. The regional population structure, negative inbreeding coefficients, and current landscape connectivity between Black Swan, Bibra and Kogolup Lakes suggest that together these populations actually represents a broader Beeliar Regional Park meta-population, and we speculate that the infinite Ne estimates could be a result of a large population and therefore not likely to suffer from genetic drift in the near future. Without increasing the sample size and recalculating the effective population size, we cannot confirm this population size. However, if the Kogoup Lake Ne is actually large then the lower bound may provide useful information about plausible Ne estimates [65, 89].”

In addition, reviewer two did not have a problem with our sample size or discussion of our infinite value.

**Line 374: This first sentence is confusing, please change it to say the lower limits of confidence intervals estimated by the Parametric and Jackknife methods were X and Y, respectively.**

We have removed Jackknife results (see above comments) so amended this sentence to only include the lower parametric CI.

**Line 391: Avoid saying “completely isolated” and use other words, maybe “…showing signs of inbreeding and genetic isolation, ultimately all….”**

In the context of this sentence, Carnac Island is completely isolated.

**Line 397: Edit for clarity: “Carnac Is. tiger snakes showed a high level of genetic distinctiveness; this is not surprising given that this population is on an isolated island. However, unexpectedly, the Carnac population also had the highest…”**

Added to lines 427-428

**Line 405: Also cite the capture/recapture study that showed larger population sizes than expected (DOI:10.1046/j.1442-9993.2002.01198.x), they suggest because of food availability.**

While this particular study showed high density and population size, it was conducted more than 20 years ago. Several of us authors have been surveying this Island for the past 20 years and, although we haven’t quantified it, found that snakes aren’t as abundant. We think abiotic changes have reduced the population size. Thus we don’t think our current findings are comparable to this recommended study.

**Lines 400-401 & 411-412 – “which ancestrally aligns with the geographically closest populations of the southern cluster (Black Swan Lake)”, I do not understand what that means?**

**The results of TESS3 suggest that the Carnac population is genetically closer to the Bibra-Kogolup-Black Swan cluster, but I do not understand what make you think that BS population is the closest one? On the compoplot with k=2, it is Bibra which shows the highest percentage of “blue” (but you should not discuss these differences as they are very low). From your results (TESS3 and the PCoA), I got that Bibra-Kogolup-Black Swan are a single genetic population.**

For clarity, we have amended the sentence to read “It is possible that the founding population was sourced from many genetically diverse populations (e.g. including the east coast subspecies), however we would expect the Carnac Island snakes to separate from our sampled populations at lower K values, and show for the geographically-closest sampled populations to show little-to-no shared ancestry with Carnac Island in our admixture plots.”

**Line 410: If you didn’t sample the source population, you might not be able to see this from the TESS3R or MDS results. Unless one of the sampled populations was more closely related to the unsampled population than the rest…it would be hard to identify - it’s therefore possible that the source snakes came from other unsampled localities.**

**At higher K values do you see evidence of mixed ancestry in the Carnac Island pop? Please remove this line or add a citation that shows that you can identify admixture from a population that is unsampled.**

We do not see any evidence of mixed ancestry in Carnac Island up to K = 7, which already splits the data into the total number of sampled a-priori populations. We feel we have made this statement clearer in the above comment.

**Line 411: Please reorganize this paragraph – the argument you are making is hard to follow as written – some specifics are outlined below. Maybe start by saying that the island has a large number of private alleles – more than you expect from de novo mutations in 90 years.**

**So, there are a few possibilities, either 1) the snakes came from other unsampled places so the mutations are ancestral, 2) the genetic mutation rate increased due to adaptation to the environment and the mutations are de novo, or 3) the snakes could be a remnant population that is much older than 90 years and the mutations are de novo.**

**I don’t think you need to discuss phenotypic plasticity, unless you want to point out that these snakes have shown phenotypic plasticity in previous studies, and epigenetics can affect mutation rates (e.g. see Ashe et al, 2021, https://doi.org/10.1098/rstb.2020.0111 and citations therein).**

Thank you for the advice. We have rearranged this paragraph and removed discussion about plasticity except in respect to epigenetics.

It now reads “Surprisingly, we found the Carnac Island population had more than three to five times the private alleles compared to the mainland populations, much more than we would expect from de novo mutations over 90 years of isolation. We propose three hypotheses: (1) the snakes originated from other unsampled populations and the mutations are ancestral; (2) the mutation rates have increased as a response to novel selection pressures [95], since the ecosystem on Carnac Island is very different to the habitat tiger snakes usually prefer on the mainland. This hypothesis could be supported by the phenotypic plasticity shown in the population [24], if epigenetically-driven plasticity has increased genome evolution [96]; or (3) the snakes are a naturally-occurring remnant population that is much older than 90 years, and the mutations are de novo. This hypothesis could be supported by Black Swan Lake’s – the geographically-closest sampled population – shared ancestry, and tiger snakes naturally occurring on the nearby Garden Island [97], historically forming the land-bridge that connected these islands to the mainland roughly 6000 years ago [98].”

**Line 414: For clarity, please edit the second phrase to, “much more than we would expect from de novo mutations over 90 years of isolation”.**

Added to line 444

**Lines 415 – 420: If you are suggesting that the genetic mutation rate increased as a response to the different habitat and prey on the island and caused the observation of more private alleles, then why are you also talking about plasticity?**

**Morphological plasticity is caused by epigenetic changes, not just phenotypic mutation rates. Although, epigenetics is related to genome evolution, so you could make that argument, and add a citation. Otherwise, please remove the sentence starting with “This hypothesis is supported by…” on line 417.**

See above comment and amended paragraph.

**Line 420: Remove the second clause of the sentence, “as phenotypic expression of a trait may simply be driven by a small portion of the entire genome”. Phenotypic plasticity is due to epigenetics, so snake populations can be phenotypically plastic and show genetic homogeneity even in the genes involved.**

See above comment and amended paragraph.

**Lines 417-423 – I do not understand what you want to say here. Phenotypic plasticity is defined as the property of a genotype to produce distinct phenotypes in response to environmental variation. It does not require mutation or selection.**

See above comment and amended paragraph.

**Line 421: Delete the sentence starting, “As a result, not all alleles are under selective or adaptive pressure…”. This is not necessary, just start with “If Carnac Island…”.**

See above comment and amended paragraph.

**Line 425 - Again, what is the generation time of tiger snakes?**

We have added this. Lines 363

**Lines 430-443 – No, this is over-interpretation. What your results show is:
- There is no gene flow between the North and the South, which could lead to genetic variation decline in the long term as the isolated northern population are at the edge of the species distribution range, but for now there is no sign of inbreeding.
- Herdsman population is isolated both from the southern and northern populations. It is also the population with the lowest body condition, but it seems to be related to the highly anthropized environment and not to the genetic variation which is similar to the other populations of the study area.
- There is a good genetic connectivity in the southern populations which belong to the same genetic cluster.**

In light of these comments, we have completely rewritten the Conclusion in a style that we believe should satisfy the reviewer, while presenting what we consider to be the major findings and interpretation of the results. The Conclusion now reads:

“Urbanisation is modifying ecosystems around the world, creating a range of stressors for wildlife living in cities. By investigating population genomic structure of species persisting in urban environments, we can gain useful information for conservation management of urban wildlife. Here, we genotyped urban and peri-urban populations of tiger snakes with the aim of understanding natural and anthropogenic influences on genomic diversity and population connectivity. We found that the major river system that runs through our urban study area has been a strong historical barrier to gene flow, resulting in the partial isolation of populations to the north of the river from the remainder of the species distribution. These northern populations also exhibited lower genomic diversity and lower body condition than southern populations, suggesting that they are most at risk of extirpation as urbanisation further encroaches upon their sensitive wetland habitats. As we expected, the populations most exposed to isolation – both geographic and urban – showed the strongest signal of inbreeding, although the maintenance of large effective population sizes appears to be driven primarily by the amount of available habitat. Together, these findings suggest that that increasing population connectivity and maximising the area of habitat in urban areas will help improve the adaptive capacity of urban wildlife. We also recommend further investigation into the genomic architecture of adaptation to urbanisation in this species, which will improve our understanding of the genetic and physiological pathways by which species adapt to urban environments.”

**Figure 1 – Please add panels number or letter to differentiate the map of Australia from the map of your study area. When you talked about the “grey shading” in the legend, I first though you were talking about the “Urban Infrastructure”. Also, it is difficult to differentiate between the different shades of green in the figure. I would suggest to simplify the figure by grouping the different vegetation classes into one class "favourable habitat" vs. urban area vs. water.**

We have simplified the figure by merging the green landscapes into one ‘natural environment’, and the two agricultural landscapes into one. We think agricultural landscape should be distinct, as tiger snakes potentially avoid it, but we don’t know for sure.

We have added panels to the figure.

**Figure 1: It is very hard to read the white text labels on the map – please change these labels to be solid black text (also the arrows). Regarding the inset map on the right, the yellow dots are too small and hard to see - please make them larger.**

We have enlarged the yellow dots and made all labels black.

**Figure 2: Please also add higher K values – also label the plots by the K value not the number of the plot: i.e. the top one should be K=2, etc.**

We have added K5-7 in a Supp figure. And fixed the K values in Fig 2.

**Figure 3 – We cannot see the bars on the figure. Also, in the legend it is not “the proportion of each individual assigned” but “the fraction of individuals genome that originates from a particular ancestral population”.**

There are too many individuals to have spacing between the bars. We have amended the legend.

**Table 3 – What is the difference between the 95% CI and the Jackknife results. What are the overall and expected r2? You must explain it in the Methods and Results sections.**

We have removed the jackknife results as they are less reliable.

We have removed the expected r2 (as it’s less informative) and included a brief description I the results and table legend.

Results now reads as “The r2 – a sample-size bias corrected value of linkage-disequilibrium across all loci pairs – was lower in the northern populations compared to the southern populations.”

Table legend now reads as “Overall r2 represents a composite measure of average linkage-disequilibrium across all pairs of loci.”

**Table 3: Please clarify the column titles in the last two columns – I think both are 95% CI, one derived from the Parametric method, the other Jackknife. If so please change the titles.**

See above comment. We have updated the CI to include parametric.
