# Supplementary material for: Bioindicator snake shows genomic signatures of natural and anthropogenic barriers to gene flow
Source: PLoS One. 2021 Oct 29;16(10):e0259124. doi: 10.1371/journal.pone.0259124 (PMC8555784; doi:10.1371/journal.pone.0259124)
Supplement: Attachment — Submitted filename: Response to Reviewers.docx [file pone.0259124.s008.docx]

**Journal requirements**

1. Ensure formatting is to journal including file naming

These are correct.

2. Provide additional location information in methods

We have included a GPS location for each site and briefly categorised sites north or south of the dividing rivers. Individual snake locations are provided in the data repository.

3. Provide data availability

We have provided the relevant accession number: Biostudies: S-BSST646 (https://www.ebi.ac.uk/biostudies/studies/S-BSST646?query=S-BSST646) and the data will be open upon publication.

4. Ensure Fig 1 complies with CC BY 4.0 license

Fig 1 contains modified layers from the Australian Bureau of Agricultural and Resource Economics and Sciences (CC BY 3.0) and IUCN Red List of Threatened Species. Both resources allow free, open distribution for scientific publications, if attributed correctly. We have attributed the resources as per their specific requirements and attached copies of their CCs.

5. Review reference list is complete and correct

These are correct.

**Reviewer 1**

I think that the results about the genetic distance among populations are over-interpreted. Although Carnac has the greatest genetic distance among other populations (0.40-0.29), the genetic distance between Carnac and Black Swan/Kogolup is moderate (0.29). Also, it is moderate when the genetic distance is compared among distant populations, for example, Yanchep and Black Swan (0.26). So, I conclude that the genetic distance among populations is not high but moderate to low, and they are not entirely isolated.

These values are not genetic distance, they are genetic differentiation (based on *G*″_ST_, an *F*_ST_ analogue). As the number of migrants strongly influences the level of differentiation, we can conclude that the population with the highest level of differentiation from other populations is likely to be the most isolated. We suggest that a differentiation value of 0.4 indicates quite strong isolation from other populations. However, we agree that we can express the results in a more communicable fashion, and have now revised the relevant paragraph in the Results section to state:

“Carnac Island was the most differentiated from other populations (pairwise G″ST = 0.29–0.40). The most geographically distant pair of sites, Yanchep and Black Swan, exhibited a moderate level of differentiation (G″ST = 0.26).”

About the investigation of the cross-entropy scree plot produced using the tess3r package, in Figure 3, I do not see the plot of K=4 in the figure. I just saw the plot of K=2 to K=3. Where is this plot (K=4)? In Figure S3, I just saw the plot of K=5 to K=7. Also, in Figure 3 and Figure S3, I suggest that the south and north lakes should be indicated in these figures because it is tough to understand what the authors present in results and what they have shown in these figures.

We accidently submitted the old figure with incorrect labelling. Fig 3 shows K2, 3 & 4. We have attached the correct version, and added additional labels clarifying the populations north/south of the rivers. We also state in the figure caption that the dashed line is the divide between north/south populations.

Also, following the cross-validation interpretation, to choose the best K, in Figure S2, the author said in Materials and Methods: “We considered ancestry coefficients for K values of 2, 3 and 4 as these values showed the largest improvements in cross-entropy, with K = 4 showing a substantial change in slope, and plotted these for each individual”.
In the manual of tess3r they mentioned: “The best choice for the K value is when the cross-validation curve exhibits a plateau or starts increasing… Be cautious about over-interpreting the value of K and the folkore around the choice of this value. Population structure is often hierarchical, and the estimation of K strongly depends on sampling and genotyping efforts. The number of genetic groups detected by ancestry estimation programs does not necessarily correspond to the number of biologically meaningful populations in the sample (Francois and Durand 2010). If I am correct, following the Figure S2 the best K in your analysis is K=4 to K=6, so why you choose K=2 to K=4?

With large genomic datasets utilising thousands of SNPs, the K value with the lowest cross-entropy is typically the same value as the overall number of sampling localities, as we have the power to identify even fine-scale differentiation among geographically close sites. This is not very informative because it doesn’t group populations into ancestral clusters or allow for inference of meaningful population structure across the landscape. A large drop in cross-entropy implies a big improvement in the explanation of genetic structure and so is useful for inference about higher levels of the hierarchy. We have now reworded the relevant paragraph of the Materials and Methods section to improve our explanation of the method, which now reads:

“The ‘tess3’ function incorporates the latitudes and longitudes of each sampled individual to account for the influence of isolation-by-distance on ancestry coefficients, with large drops or plateaus in the scree plot identifying useful values of K for inference of population genetic structure (Fig S2). We considered K values of 2, 3 and 4 most useful for describing the high-level genetic structure across the region, as these values showed the largest reductions in cross-entropy, with a plateau starting to form at K > 4. For finer-scale investigation of population structuring we also plotted ancestry coefficients of K = 5, 6 and 7 (Fig S3).”

Also, the author mentioned, “At K=3, Herdsman Lake is recovered as a distinct cluster, potentially due to isolation from a sea of urban infrastructure”. I do not see this in figure 3, I see a strong differentiation, but I see that there is gene flow with other populations. I suggest that the authors use additional analysis to see gene flow, as Ima2 and FastSimCoal. Also, the author can use Estimated Effective Migration Surface analysis (EEMS); in this analysis, you can see the landscape and the genetic structure among populations. Also, you can evaluate some geographic barriers that can explain the population structure.

Our apologies for inserting an old figure. The reviewer was thus interpreting these findings on K=2, not K=3. The new figure should improve understanding in this regard. To address the issue of visualising the pattern of population structure across the landscape, we have now included maps of the ancestry coefficients output from tess3r, for K values of 2 to 6, in the Supplementary Material. We believe this adequately allows readers to visually identify barriers to gene flow.

**Reviewer 2**

Line 2: "Top predator" probably not justifiable, nor necessary in title, or elsewhere in the MS. No evidence that tiger snakes are top or apex predators is presented in the MS or cited references. Seems to just be words added to colour the MS.

Although there is no formal study quantifying the trophic tier for tiger snakes in Australia, they are well recognised by herpetolologists - including very experienced authors of this MS – as not, or rarely, suffering predation once they attain adult size. This is supported by the lack of adult predation records in the literature. As an alternative, we propose changing the phrase in the title to “Bioindicator snake” – as there are currently six publications justifying the use of tiger snakes as bioindicators (see Lettoof, D. C. *et al*.).

Line 19: Abstract. Discovery of the much greater than predicted heterozygosity on Carnac was not an aim of the study but becomes a significant finding. It should be mentioned in the Abstract.

Our abstract is currently at the word limit and we believe it adequately summarises the key findings. Although the Carnac discovery was interesting, it was not a primary focus of the MS and we cannot describe the findings – with appropriate context – in an already filled abstract.

Line 30: why the quotes around ‘fitness’?

We have removed these quotes.

Line 95: Study Sites and Sample Collection. In this section could the authors comment on the unevenness of the sampling at the different sites? Low samples at some sites could reflect low population numbers, but other factors such as snake detectability, habitat complexity or access could also influence sampling. This unevenness, leading to potential population underestimates, could then be part of the potential set of explanations for differences between sites.
This topic could be part of the Discussion around population size estimates in lines 429-450

Kogolup, Black Swan and Carnac Island were opportunistically sampled to add sites to this study, and received less survey effort than Herdsman, Joondalup, Bibra and Yanchep. We are confident that our detection ability in catchers was the same at every site, but the lower sample size was related to survey effort. We have added a sentence in the method to clarify “Kogolup Lake, Black Swan Lake and Carnac Island were surveyed less than the other sites (a few days compared with several weeks), which resulted in lower sample sizes for these sites.”

Line 202: again, why the quotes?

We have removed these quotes.

Line 383: isolated due to urbanisation

We have amended this sentence.

Line 390: and reduced survival probability

We have added ‘reduced’.

Line 425-26: incomplete sentence: While Fraser, Ironside….habitat.

We have restructured the sentence to “While Fraser, Ironside (88) suspect that large available habitat is responsible for maintaining large population sizes in deer populations isolated by urbanisation.”

Line 439: typo: Kogolup

Amended.

Lines 466-67: Awkward sentence structure – needs rewrite.

We have rewritten to “It is possible that the founding population was sourced from many genetically diverse populations (e.g. including the east coast subspecies), if that was the case however, we would expect the Carnac Island snakes to separate from our sampled populations at lower K values, and the geographically closest sampled populations to show little-to-no shared ancestry with Carnac Island in our admixture plots.”
